# Alone you go faster, together you go farther

**DOI:** 10.1002/1878-0261.13549

**Published:** 2023-11-09

**Authors:** Nicola Aceto

**Affiliations:** ^1^ Department of Biology, Institute of Molecular Health Sciences Swiss Federal Institute of Technology Zurich (ETH Zurich) Zurich Switzerland

**Keywords:** circulating tumor cell clusters, circulating tumor cells, colonization, intravasation, metastasis

## Abstract

The metastatic process is an extraordinarily complex step‐by‐step procedure, characterized by many analogies with migratory patterns of humans or animals across our planet. The ongoing interrogation of circulating tumor cells (CTCs), caught in the act of spreading from one location to another, is revealing distinct behaviors including biological, physical, and mechanical features that impact on their likelihood to form metastasis. In this viewpoint, I will discuss some of these findings and provide a perspective on the metastatic journey, open questions and opportunities to exploit some of the most recent discoveries for the development of antimetastasis medicines.

## Graphical abstract

Metastasis is how most cancers kill. During the metastatic cascade, cancer cells acquire different properties that influence their likelihood to succeed. In this viewpoint, I summarize some of my views on metastasis‐relevant phenomena, challenges, open questions and opportunities for intervention.

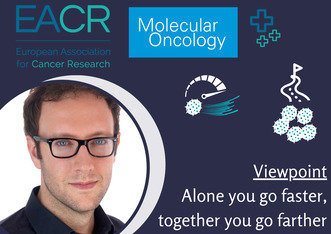

**Abbreviations**
CTC: circulating tumor cell
DTC: disseminated tumor cells
WBC: white blood cell

## 1 Introduction

The purpose of the next chapters is not to provide a comprehensive overview of the available literature related to metastasis, already offered elsewhere [1, 2, 3], but to provide a concise and thought‐provoking viewpoint on some of the critical steps involved in this process while sharing perspectives, thoughts and expectations for future discoveries. Purposely, the text is organized in three main sections, referrable to three main steps along the metastatic journey, namely intravasation, survival in circulation, and colonization.

## 2 Voluntary intravasation vs necessary escape

A question I often ask myself when thinking about the intravasation process is “why” would cancer cells do so. Is this somehow engrained in their nature (i.e., spread as much as possible), or rather, are some of them forced to intravasate, possibly because their environment in the primary site—or more precisely, in specific locations within the primary site—has become unfavorable? I recently became acquainted (or at peace, for now) with the thought that it is probably a combination of both, with the balance shifting in favor of one or the other mechanism depending on the context. There are many factors at play, such as availability of nutrients [4], expression of metastasis‐promoting genes [3, 5] and microenvironmental stimuli [1, 6] that may influence cancer cell behavior in one way or another. What is more, evidence suggests that the timing of all of the above may be dictated by systemic phenomena such as circadian rhythmicity [7, 8].

Based on the current state‐of‐the‐art, not only the “why” but also the “how” is intellectually stimulating. Cancer cells intravasate in different modalities: single cells, small homotypic and heterotypic clusters, as well as larger microemboli (larger cell groupings, typically consisting of more than 50 cells) [2, 5, 9, 10]. Each of these entities is characterized by different biological and physical/mechanical features, likely inherited from their direct neighborhood at the level of the lesion they departed from, rather than from the circulation, given the short circulation half‐life of CTCs [11]. Following this logic, one could imagine any cancerous lesion as a crowded collection of different intratumor microenvironments and neoplastic subclones with diverse characteristics, each of which with a different likelihood to intravasate and with a predisposition to do so in one modality or another. This, in my view, is a very exciting (yet unanswered) question: where exactly, within cancer lesions, do CTCs depart from? I highly anticipate a better understanding of these aspects in the years to come—enabling new strategies to anticipate (or even prevent, if possible) intravasation itself.

## 3 Fight for survival under stress

Right after intravasation is where things get rough. In circulation, cancer cells find themselves in suspension, detached from one another, under unprecedented levels of shear stress, and very visible to circulating immune cells that clearly outnumber them. As a reminder, carcinoma cells are typically used to grow in tight adherence throughout their life span. The analogy in this case, for humans or animals that would like (or be forced to) migrate, is jumping into a very rough river populated by piranhas. It is in this context that the fight for survival starts. We and others could demonstrate that when doing so, the chances for survival are greatly enhanced for multicellular aggregates [5, 9]. Particularly, both CTC‐white blood cell (WBC) clusters and homotypic CTC clusters allow themselves to keep a proliferative program switched on while in circulation, probably thanks to clustering itself, favoring both mitogenic signaling and resistance to anoikis. Clustered CTCs can count on two very idiosyncratic advantages: size and proliferation. Size is useful on the mechanical side of things, as it allows for rapid entrapment in small capillaries, leading to an interrupted circulatory flow in the entrapment site (a.k.a. a micro‐ischemic stroke). On the other hand, proliferation (and associated programs, such as stem‐like features [10]) favors survival and metastasis seeding. The most direct evidence for the relevance of CTC clustering in metastasis formation is the efficacy of anticlustering compounds in preventing metastasis in mouse models [10], which have led to ongoing clinical studies and drug development efforts [12].

## 4 Adapt, be discreet at first, then colonize

Upon arrival at the metastatic site, an entirely different game starts. Here, cancer cells quickly realize drastic differences in their microenvironment compared with their tumor of origin. The fight for survival, nutrients, life‐compatible factors continues and arguably, most metastasis pioneers are lost at this stage. There is a certain consensus in the field that a temporary exit from the cell cycle (e.g., referred to as dormancy) might be beneficial for survival. While it seems against the nature of cancer cells themselves to voluntarily enter dormancy, the most likely scenario is that dormancy is “imposed” upon them by extrinsic signals, for instance by the scarcity of mitogenic stimuli in the new microenvironment as well as the patrolling activities of antitumor WBCs [13]. This somehow allows cancer cells to persist in stealth mode, until reactivation occurs. In this setting, “when” and “what” lead to this reactivation is an outstanding question that deserves further investigation. Also, how single and clustered disseminated tumor cells engage with dormancy and awakening is currently unclear. As soon as cancer cells exit dormancy, available data support similar to much higher (explosive) proliferation rates compared with their tumor of origin [13]. Clearly, the mission is now to colonize as widely and as quickly as possible and eventually achieve further, metastasis‐to‐metastasis dissemination [14, 15]. Needless to say, while this is extraordinarily challenging, further understanding of the biological features of dormancy, re‐awakening, and adaptation to new anatomical sites is likely to enable improved adjuvant approaches in the clinic.

## 5 Concluding remarks

When seen all together, while numerous questions remain, the last decades have witnessed important advances in our understanding of the processes that characterize the accomplishment of metastasis. Several targets and mechanisms have been proposed, many of which are now worthy and mature enough to be tested in the clinic. In this context, however, approved endpoints are not necessarily designed to specifically address metastasis, but they are rather tuned for demonstrating tumor killing activity. It is only recently (as of 2021) that the FDA has released recommendations/guidance for using metastasis‐free survival endpoints in clinical studies, likely recognizing the importance of metastasis‐prevention strategies that are expected to arise in the years to come.

## Conflict of interest

N.A. is cofounder and member of the board of PAGE Therapeutics AG, Switzerland, listed as inventor in patent applications related to CTCs, a paid consultant for companies with an interest in liquid biopsies, and a Novartis shareholder.
